# Adenovirus-vectored T cell vaccine for hepacivirus shows reduced effectiveness against a CD8 T cell escape variant in rats

**DOI:** 10.1371/journal.ppat.1009391

**Published:** 2021-03-18

**Authors:** Alex S. Hartlage, Piyush Dravid, Christopher M. Walker, Amit Kapoor

**Affiliations:** 1 Center for Vaccines and Immunity, Abigail Wexner Research Institute at Nationwide Children’s Hospital, Columbus, Ohio, United States of America; 2 Medical Scientist Training Program, College of Medicine and Public Health, The Ohio State University, Columbus, Ohio, United States of America; 3 Department of Pediatrics, College of Medicine and Public Health, The Ohio State University, Columbus, Ohio, United States of America; The University of Chicago, UNITED STATES

## Abstract

There is an urgent need for a vaccine to prevent chronic infection by hepatitis C virus (HCV) and its many genetic variants. The first human vaccine trial, using recombinant viral vectors that stimulate pan-genotypic T cell responses against HCV non-structural proteins, failed to demonstrate efficacy despite significant preclinical promise. Understanding the factors that govern HCV T cell vaccine success is necessary for design of improved immunization strategies. Using a rat model of chronic rodent hepacivirus (RHV) infection, we assessed the impact of antigenic variation and immune escape upon success of a conceptually analogous RHV T cell vaccine. Naïve Lewis rats were vaccinated with a recombinant human adenovirus expressing RHV non-structural proteins (NS)3-5B and later challenged with a viral variant containing immune escape mutations within major histocompatibility complex (MHC) class I-restricted epitopes (escape virus). Whereas 7 of 11 (64%) rats cleared infection caused by wild-type RHV, only 3 of 12 (25%) were protected against heterologous challenge with escape virus. Uncontrolled replication of escape virus was associated with durable CD8 T cell responses targeting escaped epitopes alone. In contrast, clearance of escape virus correlated with CD4 T cell helper immunity and maintenance of CD8 T cell responses against intact viral epitopes. Interestingly, clearance of wild-type RHV infection after vaccination conferred enhanced protection against secondary challenge with escape virus. These results demonstrate that the efficacy of an RHV T cell vaccine is reduced when challenge virus contains escape mutations within MHC class I-restricted epitopes and that failure to sustain CD8 T cell responses against intact epitopes likely underlies immune failure in this setting. Further investigation of the immune responses that yield protection against diverse RHV challenges in this model may facilitate design of broadly effective HCV vaccines.

## Author summary

The hepatitis C virus is one of the leading causes of chronic liver disease and cancer worldwide. A vaccine is not yet available and the first phase II clinical trial in humans using a T cell-based immunization strategy recently failed to prevent chronic infection in high risk individuals for unclear reasons. In this study we evaluated how immune escape mutations at major histocompatibility complex (MHC) class I-restricted viral epitopes influence the effectiveness of an adenoviral-vectored T cell vaccine in a rat model of chronic HCV-related rodent hepacivirus infection, currently the only animal model available for evaluation of HCV vaccine strategies. We show that vaccine efficacy is markedly diminished when challenge virus contains naturally-acquired escape mutations at dominant MHC class I-restricted viral epitopes that render a subset of vaccine-generated CD8 T cell responses ineffective. We also identify CD4 T cell help as a critical correlate of vaccine success against heterologous virus challenge. Our results have important implications for human vaccination programs that aim to induce broad protective immunity against heterogeneous HCV strains.

## Introduction

Hepatitis C virus (HCV) chronically infects 71 million people worldwide and contributes to 399,000 deaths annually[1]. Despite the advent of all oral, direct-acting antiviral (DAA) regimens that cure most infections, there are significant barriers to HCV eradication that necessitate development of a preventative vaccine. These include low rates of infection diagnosis, high cost of treatment, and emergence of drug-resistant strains[2,3]. In addition, DAA cure does not afford protective immunity against HCV reinfection[4], complicating the elimination of HCV within high-risk patient groups.

The first and only clinical trial to evaluate HCV vaccine efficacy in humans has recently concluded (NCT01436357). The investigational regimen consisted of sequential doses of chimpanzee adenoviral and modified vaccinia virus Ankara vaccines encoding HCV non-structural (NS) proteins, with a primary goal of inducing protective T cell immunity. Despite encouraging preclinical data[5,6] and a large body of evidence suggesting a critical role for T cells in immunity to HCV[7,8], no reduction in the overall incidence of chronic infection was observed[9]. Failure of this highly anticipated trial highlights a concerning gap in our understanding of protective immune responses to HCV and a need to identify factors that influence HCV vaccine efficacy.

A principal challenge for HCV vaccine development is overcoming the virus’ enormous genetic diversity[10]. HCV is composed of at least 7 major genotypes and more than 80 subtypes that differ considerably in amino acid sequence[11]. Additionally, the error-prone nature of HCV’s RNA-dependent RNA polymerase enables generation of diverse mutant progeny that can escape recognition by protective B and T cell responses[8]. This inter- and intra-strain diversity suggests that a broadly-reactive vaccine response will be necessary to protect against most if not all HCV variants. Indeed, a primary rationale for the selection of HCV NS proteins as a vaccine immunogen is their relative conservation amongst HCV strains. This property is reflected in the capacity for NS proteins from HCV genotype 1b to induce human T cell responses recognizing multiple heterologous strains[6]. Nevertheless, suboptimal recognition of infecting virus due to partial immunogen mismatch could significantly undermine the efficacy of an HCV vaccine.

Because of the NIH’s present moratorium on chimpanzee research, there are currently limited avenues to conduct mechanistic testing of candidate HCV vaccines. Recent discovery of an HCV-like rodent hepacivirus (RHV) in rats, however, has enabled development of a small animal challenge model[12–15]. The benefits of this surrogate system include ease of manipulation, immune competency, and high susceptibility to chronic infection. Although RHV differs substantially from HCV in amino acid sequence, similarities in genetic structure and organization, liver tropism, and DAA susceptibility make it highly relevant for HCV study[14,16,17]. Indeed, two recent studies, conducted in inbred and outbred rat strains, showed that recombinant adenoviral vectors encoding the RHV NS3-5B proteins reduce the incidence of persistent infection after homologous challenge[13,15]. Prolonged or persistent RHV infection after antibody-mediated depletion of CD8 or CD4 T cells, respectively, established a critical role for cellular immunity in vaccine protection[13]. Because natural variants of RHV have not yet been discovered, vaccine efficacy against infection by heterologous virus, particularly that which is mismatched at dominant T cell epitopes, is unknown.

In the present study we describe the generation of an RHV variant expressing immune escape mutations within major histocompatibility complex (MHC) class I-restricted epitopes that are dominant targets of CD8 T cell immunity in rats. Because this variant can approximate a heterologous challenge, we examined host-virus dynamics and protective immunity upon passage into rats immunized with a T cell-inducing adenoviral vector. Our findings have important relevance for HCV vaccine development and also provide unique insight into the role of mutational escape as a mechanism underlying hepacivirus persistence.

## Results

### Vaccine selection of an RHV escape variant

**Fig 1:**
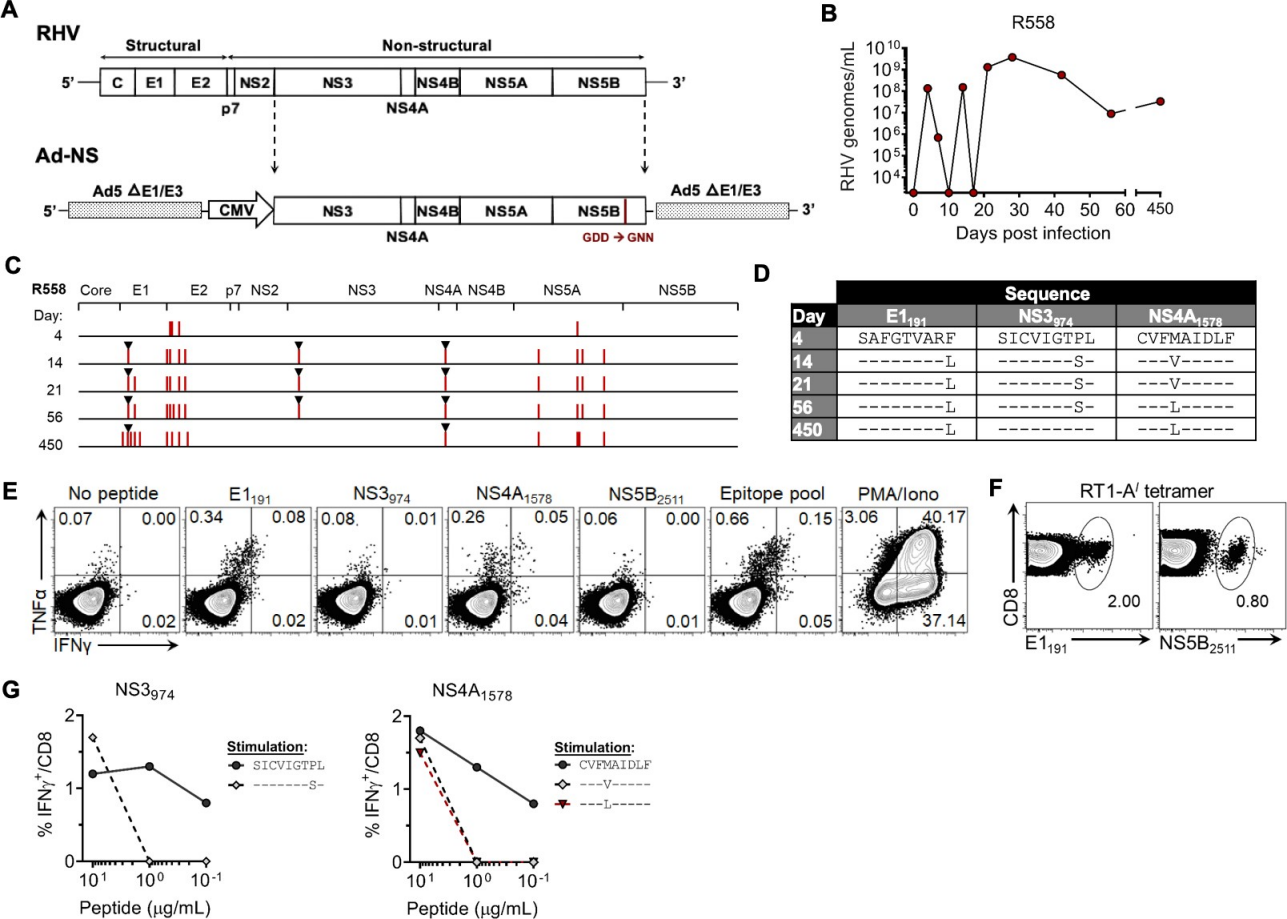
Evolution of R558 immune escape variants. Rat R558 was immunized with Ad-NS and assessed three weeks later for protection against homologous challenge. **(A)** Schematic outline of recombinant Ad-NS vector expressing the RHV NS3-5B proteins under control of a high-expression CMV promoter. The GDD catalytic site of the NS5B RNA polymerase was mutated to GNN to disrupt in vivo replication activity. **(B)** Course of RHV viremia. Limit of detection of RT-PCR assay was 1875 genomes/mL. **(C)** Sequence evolution of RHV polyprotein as determined by direct PCR sequencing. Vertical red lines indicate location of consensus amino acid substitutions. Black triangles mark substitutions arising within known RT1-A^*l*^-restricted class I epitopes. **(D)** Sequence identities of E1_191_, NS3_974_, and NS4A_1578_ escape variants. **(E)** Flow plots showing frequency of intrahepatic CD8 T cells from R558 at day 450 post infection producing intracellular IFNγ or TNFα after 5-hr stimulation with pooled class I/II (5 ug/mL each; Table 1) or individual epitopes (10 μg/mL). The response following stimulation with PMA and ionomycin is shown as a positive control. **(F)** Flow plots showing frequency of intrahepatic CD8 T cells from R558 at day 450 post infection that bind RT1-A^*l*^ tetramer specific for the RHV E1_191_ or NS5B_2511_ epitopes. **(G)** Frequency of CD8 T cells from immune rat producing IFNγ upon stimulation with titrated concentrations of wild-type or variant NS3_974_ and NS4A_1578_ peptides.

We recently demonstrated that vaccination of Lewis rats with a recombinant human adenovirus expressing the RHV NS3-5B proteins (Ad-NS; Fig 1A) confers partial protection against persistent infection[13]. In a single vaccinated rat (R558), breakthrough viremia following initial control of RHV infection coincided with emergence of non-synonymous mutations within two RT1-A^*l*^-restricted class I epitopes (NS3_974_, NS4A_1578_). A methionine to valine substitution at position 1581 (M1581V) resulted in diminished peptide recognition by NS4A_1578_-specific CD8 T cells, indicating viral immune escape at this epitope[13]; escape at the NS3_974_ epitope was not confirmed. Since non-synonymous evolution of RHV class I epitopes does not occur after naïve infection due to lack of functional CD8 T cell immunity[17], appearance of these mutations was a direct consequence of a vaccine-induced response.

Here, we provide an updated course of viremia for rat R558 along with complete polyprotein sequencing data. As initially reported[13], RHV viremia underwent large swings in magnitude (>10,000-fold) during days 7–21 post infection (p.i.) before reaching relatively stable titers (10^7^−10^10^ genomes/mL) lasting >1 yr (Fig 1B). Direct PCR sequencing of rebound virus at day 14 p.i. following initial control of viremia revealed a total of 11 amino acid substitutions, most occurring within NS5A and a presumptive hypervariable region near the N-terminus of the E2 glycoprotein (Figs 1C and S1). The P981S and M1581V substitutions arising within NS3_974_ and NS4A_1578_ epitopes, respectively,[13] were confirmed (Fig 1D). The previously described F199L immune escape substitution occurring within the RT1-A^*l*^-restricted E1_191_ epitope[17] was also found (Fig 1D); selection of this variant is particularly notable since envelope glycoproteins were not encoded by vaccine and cytokine-producing E1_191_-specific CD8 T cells are not generated during naïve infection[17]. This suggests that presence of vaccine-generated anti-NS3-5B immunity facilitated the functional maturation of anti-E1 responses that were primed by infection. Sequences of all other known RHV class I epitopes (Table 1) were intact (Fig 1C). The functional significance of mutations occurring outside of class I epitope regions was not determined, but could be compensatory.

**Table 1:** RHV class I and II epitopes^a^.

Polyprotein location^*b*^	Amino acid sequence	MHC class I/II
E1_191-199_	SAFGTVARF	I
E2_439-456_	SAGWTNLACYGQKGPFLP	I
NS3_974-982_	SICVIGTPL	I
NS3_1299-1316_	IQKGRHLIFQTSKSHCDN	II
NS3_1425-1442_	IVPDACIYEAFDSGLAYF	II
NS3_1446-1463_	PAEVATHLSFYHNQVGLP	II
NS3_1481-1498_	YVQSNYLEMMKNRVDSYT	II
NS3_1487-1495_	YTYLYAAQY	I
NS3_1502-1519_	AAQYQLAKAEGAMAPNDN	II
NS4A_1578-1586_	CVFMAIDLF	I
NS4B_1747-1764_	MVGHAFLTYGSATSACLV	II
NS5A_2248-2265_	MELLREYETSNDHVPKED	II
NS5B_2486-2503_	SGKTEIVKTLYSKLEEGI	II
NS5B_2511-2519_	CVMPKIETF	I
NS5B_2552-2569_	VEKMVLGQIGPKTVKAVC	II
NS5B_2559-2576_	QIGPKTVKAVCGDAYGFV	I

^*a*^Epitopes were identified in ref (13,17) and are RT1-A^*l*^ (class I) or RT1-B/D^*l*^ (class II) restricted according to the MHC background of Lewis rats

^*b*^Amino acid position according to the start of the RHV polyprotein (accession no. KX905133; protein cleavage sites predicted in ref (14)

The F199L substitution persisted throughout chronic infection while the M1581V was replaced by a novel M1581L substitution at day 56 p.i. (Fig 1D), possibly due to enhanced *in vivo* viral fitness[18] or escape from *de novo* T cell responses generated against the mutated epitope. Presence of these substitutions at day 450 p.i. was associated with cytokine-producing CD8 T cells specific for the E1_191_ and NS4A_1578_ epitopes (Fig 1E). In contrast, the P981S substitution reverted to wild-type sequence by day 450 p.i. (Fig 1D), likely due to functional loss of NS3_974_-specific CD8 T cells (Fig 1E) via exhaustion or deletion. Interestingly, stimulation with the dominant NS5B_2511_ epitope, which was intact throughout infection, failed to elicit a cytokine response (Fig 1E) despite moderate levels of tetramer-positive cells in liver (Fig 1F), which is consistent with a state of exhaustion driven by persistent antigenic stimulation. Importantly, testing of wild-type and variant peptides in CD8 T cell recognition assays confirmed P981S and M1581L as immune escape substitutions (Fig 1G). These data suggest that vaccine failure in rat R558 was due, at least partially, to loss of immune recognition facilitated by selection of CD8 T cell escape mutants.

### Reversion of CD8 T cell escape mutations after passage into naïve rats

**Fig 2:**
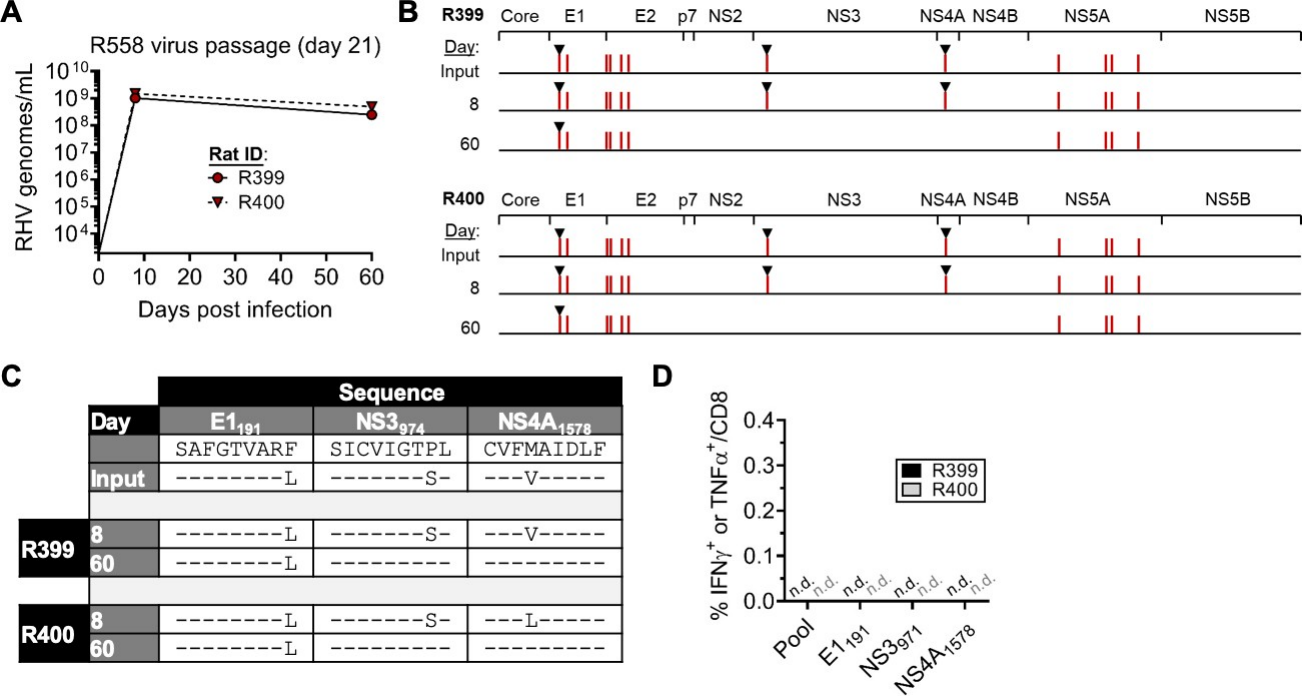
Stability of R558 escape mutations after passage into naïve rats. Unvaccinated rats R399 and R400 were challenged with 10^6^ genomes of R558 virus collected at 21 days post infection. **(****A)** Course of RHV viremia. Limit of detection of RT-PCR assay was 1875 genomes/mL. **(B)** Sequence evolution of RHV polyprotein as determined by direct PCR sequencing. Vertical red lines indicate location of consensus amino acid substitutions. Black triangles mark substitutions arising within known RT1-A^*l*^-restricted class I epitopes. (C) Sequence identities of E1_191_, NS3_974_, and NS4A_1578_ escape variants. **(D)** Frequency of intrahepatic CD8 T cells producing IFNγ or TNFα after 5-hr stimulation with pooled class I/II (5 ug/mL each) or individual epitopes (10 μg/mL). Response was determined by intracellular cytokine staining assay. n.d., not detected.

To assess the stability of these immune escape substitutions in absence of strong CD8 T cell selection pressure, we passaged RHV recovered from R558 into two naïve rats. Circulating virus from day 21 p.i. (hereafter referred to as “escape virus”) was selected for transfer since at least three MHC class I epitopes were mutated at this timepoint (Fig 1B). High levels of viremia (~10^9^ genomes/ml) were present by day 8 p.i. and persisted for several months without substantial change in magnitude (Fig 2A). Remarkably, all non-synonymous mutations were present at day 8 p.i., including those occurring outside of known RHV class I epitopes (Fig 2B and 2C). By day 60 p.i., the P981S and M1581V substitutions reverted to wild-type sequence, while all other non-synonymous changes, including the F199L substitution, remained (Fig 2B and 2C). Consistent with previous data[13,15,17], functional RHV-specific CD8 T cell responses were lacking at this timepoint (Fig 2D). Persistence of the F199L substitution without apparent immune pressure suggests that it does not confer a significant cost to *in vivo* viral fitness or is sufficiently stabilized by co-occurring compensatory mutations (Fig 2B). These data suggest that a strong RHV-specific CD8 T cell response is necessary to maintain the P981S and M1581V mutations within replicating escape virus.

### Vaccine efficacy against escape virus

**Fig 3:**
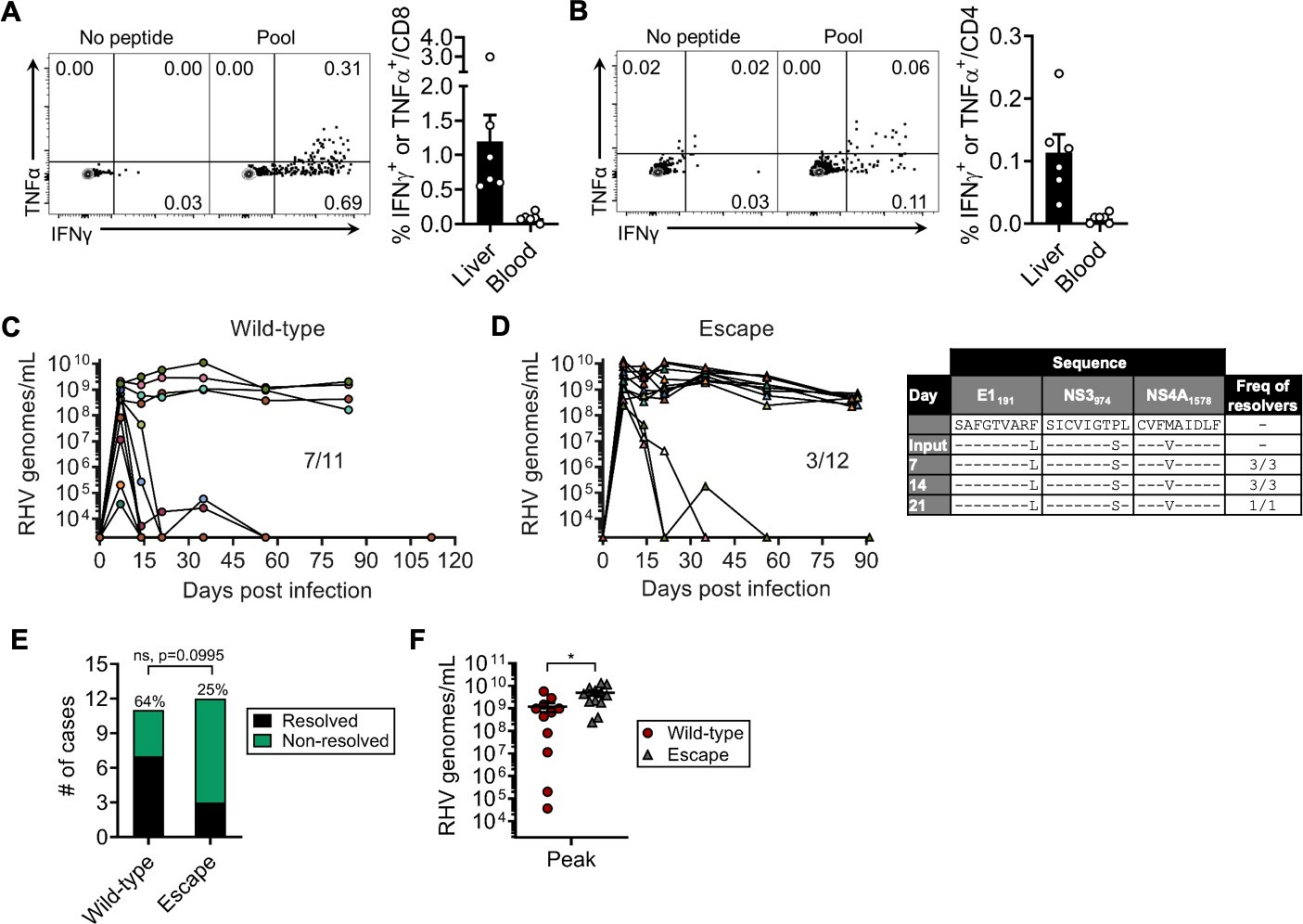
Vaccine efficacy against wild-type versus escape virus. Naïve rats received two intramuscular doses of Ad-NS (5 x 10^8^ infectious units) separated three weeks apart. Three weeks after boost, rats were euthanized for assessment of T cell immunity or challenged with 10^6^ genomes wild-type or R558 escape virus (day 21). **(A)** Representative flow plots (left panel) and summary data (right panel; mean ± SEM) showing frequency of intrahepatic or blood CD8 T cells that stain positive for IFNγ or TNFα after 5-hr stimulation with pooled class I/II epitopes (5 ug/mL each; Table 1). **(B)** Representative flow plots (left panel) and summary data (right panel; mean ± SEM) showing frequency of intrahepatic or blood CD4 T cells that stain positive for IFNγ or TNFα after 5-hr stimulation with pooled class I/II epitopes (5 ug/mL each; Table 1). **(C)** Course of RHV viremia after challenge by wild-type virus. **(D)** Left panel, course of RHV viremia after challenge with escape virus. Limit of detection of RT-PCR assay was 1875 genomes/mL. Right panel, sequence evolution of E1_191_, NS3_974_, and NS4A_1578_ epitopes in rats that resolved escape virus infection. Consensus sequences were determined by direct PCR sequencing. Frequency of rats containing virus with the indicated sequences are shown. **(E)** Comparison of infection outcome between challenge groups. ns, not significant as determined by Fisher’s exact test. **(F)** Comparison of RHV viremia at day 7 post infection (mean ± SEM). *, p<0.05 as determined by Student’s t-test.

We next sought to determine whether virus containing the aforementioned escape mutations is less sensitive to a T cell-based vaccine expressing wild-type RHV antigen. We vaccinated rats with Ad-NS, which protects majority of animals from chronic infection following homologous challenge[13]. A booster dose was given after three weeks in an effort to enhance protective efficacy[15]. Three weeks after boosting rats were challenged with wild-type RHV or escape virus at equal dose. Consistent with prior findings[13], this regimen induced moderate frequencies of cytokine-producing CD8 T cells in liver (Fig 3A), with low levels of CD4 T cell help (Fig 3B). Overall, 7 of 11 (64%) rats challenged with wild-type RHV were protected against persistent infection after establishment of acute viremia (Fig 3C). As previously reported[13], viremia was terminated or underwent substantial decline during the first three weeks of acute resolving infection. In contrast, only 3 of 12 (25%) rats controlled infection caused by escape virus (Fig 3D, left panel). Importantly, clearance of escape virus in this group was not facilitated by reversion of variant class I epitopes to wild-type sequence (Fig 3D, right panel). In both challenge groups, acute non-resolving infection was characterized by stable high-level viremia (Fig 3C and 3D). Despite a clear trend towards diminished immunity against escape virus (64% vs 25%), the difference in infection outcome between challenge groups was marginally insignificant (Fig 3E; p = 0.0995). There was, however, a significant increase in peak viremia in rats challenged with escape virus (Fig 3F), indicating less effective control of viral replication.

### T cell immunity and viral evolution during RHV persistence

**Fig 4:**
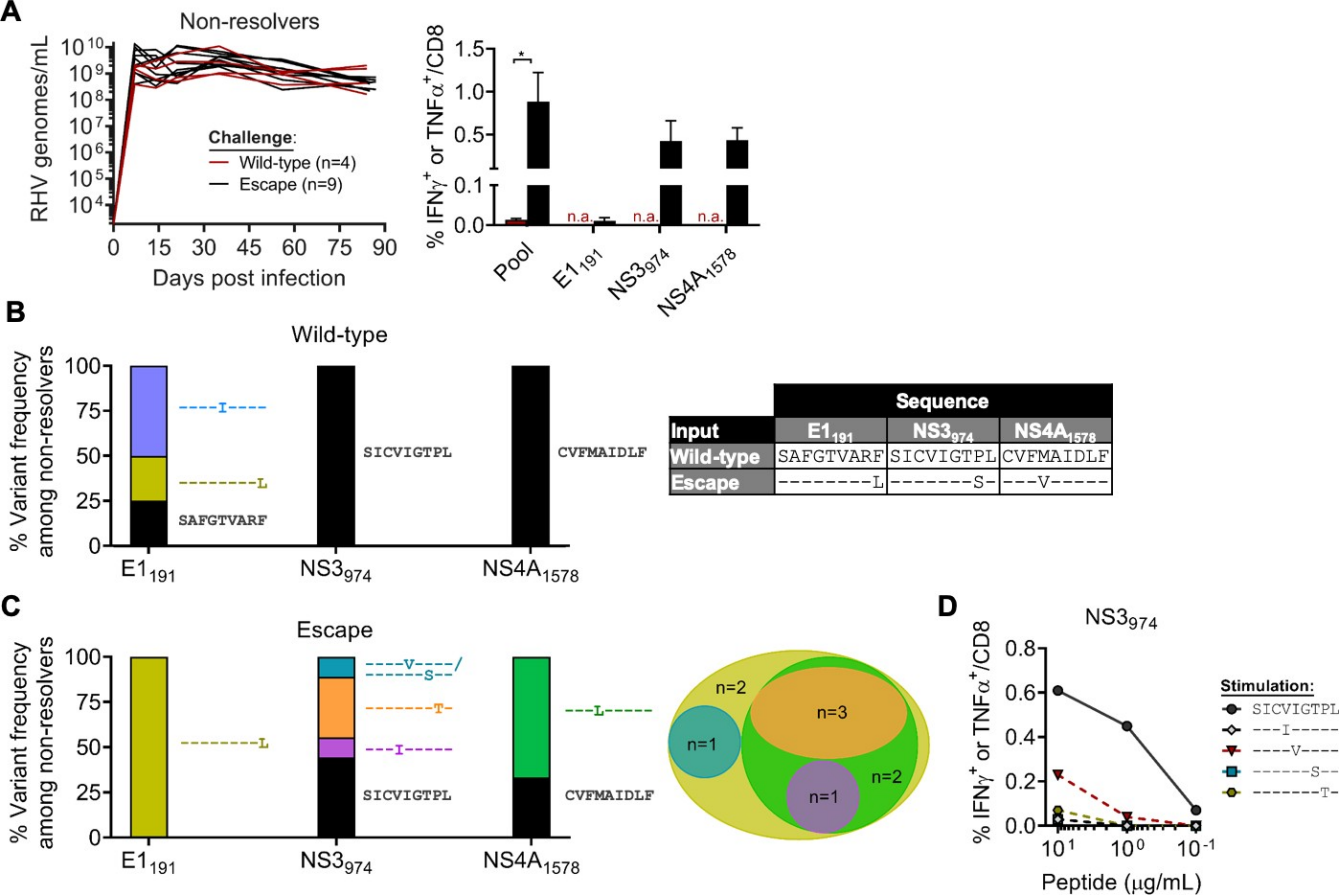
T cell responses and viral evolution in infection non-resolvers. Vaccinated rats that failed to control wild-type or escape virus infection were analyzed for T cell immunity and viral mutations (E1_191_, NS3_974_, NS4A_1578_) at day 84–91 post infection. **(A)** Left panel, course of RHV viremia. Limit of detection of RT-PCR assay was 1875 genomes/mL. Right panel, frequency of intrahepatic CD8 T cells producing IFNγ or TNFα (mean ± SEM) after 5-hr stimulation with pooled class I/II (5 ug/mL each; Table 1) or individual epitopes (10 μg/mL). Response was determined by intracellular cytokine staining assay. n.a., not assessed. **(B)** Epitope variant frequencies within wild-type challenge group. **(C)** Left panel, epitope variant frequencies within escape virus challenge group. Presence of wild-type epitope indicates sequence reversion. Consensus amino acid identities were determined by direct PCR or clonal sequencing. Right panel, venn diagram illustrating the proportion of rats with the indicated epitope variants. **(D)** Frequency of CD8 T cells from immune rat producing IFNγ or TNFα upon stimulation with titrated concentrations of wild-type or variant NS3_974_ peptides.

To determine whether viral immune escape contributed to vaccine failure within each challenge group, we analyzed intrahepatic T cell responses and class I epitope sequences at time of study termination (day 84–91 p.i.). Despite no apparent difference in the course of chronic viremia between challenge groups (Fig 4A, left panel), there was considerable difference in the frequency of cytokine-producing CD8 T cells that recognized wild-type RHV antigens. Indeed, rats challenged with escape virus showed durable, high magnitude responses against a pool of class I and II epitopes (Table 1), whereas similar activity was lacking in the wild-type challenge group (Fig 4A, right panel). This apparent lack of T cell pressure within the wild-type challenge group was overall reflected at the viral sequencing level. The escape-prone NS3_974_ and NS4A_1578_ epitopes, which were targeted by vaccination, were intact in all animals (Fig 4B). The E1_191_ epitope, by contrast, showed some diversification. Virus from a single rat contained the F199L substitution, while two others expressed a novel T196I variant; since these substitutions were not accompanied by strong CD8 T cell selection pressure similar to R558 (Fig 1D), it is possible that these mutations were driven by an anti-E1 antibody response or earlier T cell activity.

By comparison, class I epitope sequences in the escape virus challenge group were dynamic. The P981S substitution within NS3_974_ reverted to wild-type in 4 of 9 (44.4%) rats and was replaced by one of four novel substitutions (V977I, I978V, T980S, P981T) in remaining animals (Fig 4C, left panel); these novel variants were confirmed as immune escape substitutions (Fig 4D). The M1581V substitution within NS4A_1578_ was replaced by M1581L in 5 of 9 (55.6%) rats as originally occurred in R558 and reverted to wild-type sequence in all others (Fig 4C, left panel). Selection of new escape mutations in NS3_974_ and NS4A_1578_ was consistent with there being durable T cell pressure against these regions (Fig 4A, right panel). Similar to naïve passage, the F199L substitution persisted in all rats (Fig 4C) despite the lack of an E1_191_-specific functional response (Fig 4A, right panel). Overall, 7 of 9 (77.8%) rats were persistently infected with virus expressing non-synonymous mutations in either NS3_974_ or NS4A_1578_ that were not present in the escape virus inoculum (Fig 4C, right panel), suggesting continued viral evolution resulting from ongoing CD8 T cell selection pressure.

Taken together, these data strongly suggest that lack of control of escape virus in most rats was due to an inability to recognize mutated class I epitopes, particularly NS3_974_ and NS4A_1578_ that were targeted by vaccination. By contrast, persistence of wild-type virus was associated with overall absent T cell responses and no clear evidence of viral immune escape from vaccine-induced immunity.

### T cell responses associated with protection against escape virus

**Fig 5:**
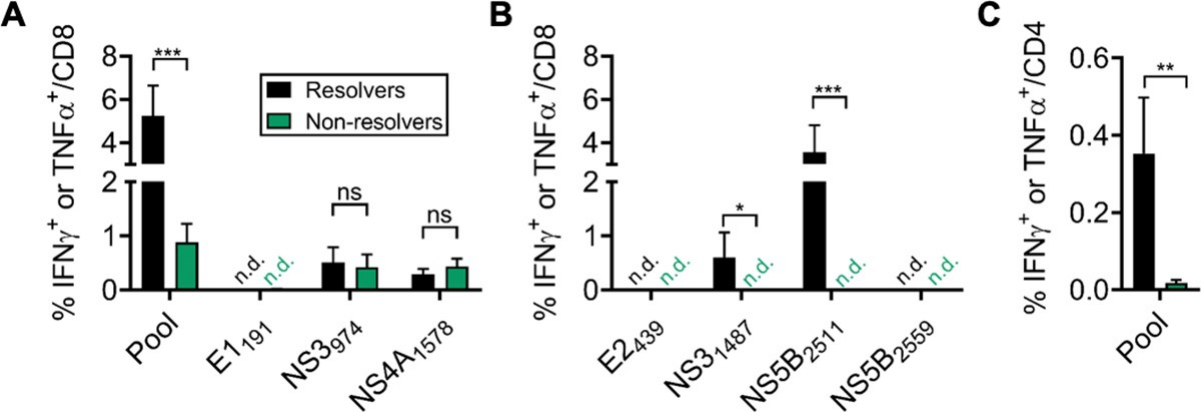
RHV-specific T cell responses after clearance or persistence of escape virus. Intrahepatic leukocytes from rats challenged with R558 escape virus (day 21) were stimulated for 5-hr with pooled RHV class I/II epitopes (5 ug/mL each) or individual peptides (10 ug/mL) in an intracellular cytokine staining assay. Responses were assessed at day 84–91 post infection. **(A,B)** Frequency of CD8 T cells producing IFNγ or TNFα (mean ± SEM). Cryopreserved cells were used for stimulation of responses in panel (B). **(C)** Frequency of CD4 T cells producing IFNγ or TNFα (mean ± SEM). ns, not significant; *, p<0.05; **, p<0.005, ***, p<0.001 as determined by Student’s t-test.

Next, to identify immune mechanisms facilitating control of escape virus in minority of rats, we compared intrahepatic T cell responses between infection outcome groups (resolvers vs. non-resolvers) at day 84–91 p.i. No significant difference was found in the frequency of cytokine-producing CD8 T cells specific for the escape-prone NS3_974_ or NS4A_1578_ epitopes that were mutated in escape virus; responses against the E1_191_ epitope were absent in both groups (Fig 5A), possibly to due to failed priming by variant peptide. However, a significantly higher frequency of cytokine-producing cells was found in resolvers following stimulation with a pool of peptides containing seven class I epitopes (Table 1 and Fig 5A), suggesting greater functional breadth. Further analysis of cryopreserved samples indeed revealed significant enrichment for CD8 T cells targeting the NS5B_2511_ and, less strongly, NS3_1487_ epitopes (Fig 5B). This preservation of CD8 T cell functionality against intact epitopes in resolvers was further accompanied by enhanced recall of RHV-specific CD4 T cell immunity (Fig 5C). These findings suggest that elimination of escape virus was related to the capacity to sustain CD8 T cell responses against intact epitopes, a feature possibly supported by presence of CD4 T cell help.

### Resolution of wild-type RHV infection yields protective immunity to escape virus

**Fig 6:**
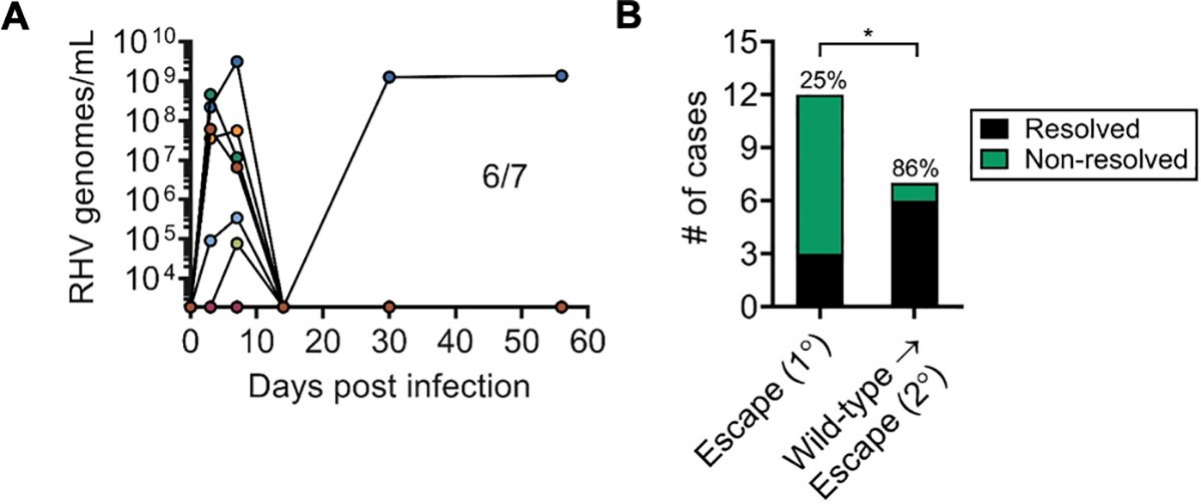
Secondary immunity to escape virus after clearance of wild-type RHV infection. Rats that resolved primary infection by wild-type RHV were challenged with 10^6^ genomes R558 escape virus (day 21). Rechallenge occurred ≥56 days after termination of first infection. **(A)** Course of RHV viremia. Limit of detection of RT-PCR assay was 1875 genomes/mL. **(B)** Comparison of infection outcome between primary and secondary challenge groups. **, p<0.005 as determined by Fisher’s exact test.

Resolution of wild-type RHV infection appears to boost vaccine-induced CD4 T cell help and induce functional CD8 T cell responses to epitopes not encoded by adenoviral vaccine[13,17]. This broadening and enhancement of RHV-specific immunity may confer protection against heterologous strains, as observed for HCV[19,20]. We therefore tested whether rats that previously controlled wild-type RHV infection were protected against secondary challenge with escape virus. Expectedly, 6 of 7 (86%) rats were protected from persistent infection after experimental challenge (Fig 6A). All rats, except for a single aviremic animal, developed variable levels of viremia that were controlled by day 14 p.i. A single rat, however, developed breakthrough viremia at day 30 p.i. Despite this, the overall rate of protection was significantly increased compared to primary vaccine challenge (Fig 6B; 86% vs 25%). This finding demonstrates that, although vaccination with Ad-NS induces weak protection against escape virus, achieving a high level of protection is feasible in concept.

## Discussion

Vaccination against HCV using T cell-based viral vectors targeting non-structural proteins recently failed to prevent chronic infection in a high-risk population of injection drug users[9]. There is presently no explanation for why this trial failed, an urgent question given the strong preclinical data supporting its potential efficacy[5,6,13,15,21]. Because of HCV’s extraordinary diversity, one plausible mechanism is insufficient genetic overlap between vaccine immunogen and the infecting HCV strain, hindering viral recognition by vaccine-established T cell responses. Here, we directly evaluated this risk using the RHV rat model which closely mimics HCV infection and viral persistence.

Our results indicate that a virally-vectored RHV T cell vaccine, which is principally analogous to the human approach and generally efficacious[13], is less effective when challenge virus contains immune escape mutations that render it poorly recognizable to vaccine-elicited CD8 T cells. Importantly, these mutations arose naturally during persistent infection of rat R558 and thus had a high probability of disrupting immune protection in new vaccine recipients. Although not truly representative of the type of heterologous exposure events expected to occur after HCV vaccination, these results provide a conceptual approximation of what might ensue when vaccine-elicited CD8 T cell responses are effectively narrowed due to absence of recognizable epitopes in infecting virus. The major advantage of this work, in comparison to earlier chimpanzee studies assessing heterologous immunity conferred via T cell-based vaccines[5,22], is that viral persistence is a universal feature of rat RHV infection in absence of vaccination, enabling uncomplicated analysis of infection outcomes.

These data also provide insight into mutational escape as a causal mechanism of hepacivirus persistence. Persistence of acute HCV infections is strongly associated with emergence of escape mutations in dominant MHC class I epitopes in chimpanzees[23,24] and humans[25–27]. However, direct evidence to support CD8 T cell escape as a cause rather consequence of HCV persistence has been difficult to establish in absence of robust animal models with identical MHC backgrounds. Because the restricting RT1-A^*l*^ allele is conserved amongst the Lewis strain, we were able to directly address the impact of viral escape on infection outcome via passage of escape virus into uninfected individuals. The higher persistence rate of escape virus in vaccinated rats illustrates that these mutations do negatively influence vaccine control of infection. Similar results might be expected for HCV as well given similar experimental constraints.

It is necessary to acknowledge that, despite a sizable reduction in vaccine efficacy against escape virus, this difference was marginally insignificant in comparison to wild-type RHV challenge. Therefore, interpretation of our results should be tempered. However, several lines of evidence suggest that infection initiated by escape virus did indeed result in fundamentally altered patterns of RHV immunity and viral persistence. First, there was overall weaker control of infection initiated by escape virus as illustrated by significantly increased peak viremia irrespective of outcome. This was presumably due to poorer viral recognition by vaccine-established CD8 T cell responses. Second, failure to eliminate escape virus was associated with durable CD8 T cell responses targeting escape-prone epitopes, specifically NS3_974_ and NS4_1578_ that were targeted by vaccine. These responses were actively maintaining viral variation at these epitopes, as evidenced by emergence of novel escape substitutions. It is unclear whether these mutations emerged as a consequence of viral evolution to more a fit state[28] or because *de novo* T cell responses specific to the original escape variant were generated[29]. Regardless of the reason, a similar phenomenon was absent from rats that failed to control wild-type RHV infection, suggesting a predominantly different mechanism of vaccine failure (e.g. immune exhaustion) in this specific challenge setting.

Our results also shed light on the immune responses required to control a heterologous infection. Indeed, we found that vaccine-mediated clearance of escape virus, although infrequent, significantly correlated with sustained CD8 T cell cytokine activity against intact epitopes in NS3 and, in particular, NS5B. These responses were absent from persistently infected rats despite similar levels of functional CD8 T cells specific to escaped epitopes. Since CD8 T cell immunity was not assessed prior to infection, it is conceivable that responses to intact epitopes were not adequately primed by vaccine in these animals, thus permitting uncontrolled viral replication. However, given the strong CD8 T cell-inducing potential of the adenovirus vector utilized here, a more plausible scenario is that vaccine responses were deleted and/or became functionally exhausted during progression to persistent infection as is typical of HCV[7]. Indeed, acquisition and later reversion of the P981S escape mutation in virus from rat R558 suggests that relaxation of virus-specific CD8 T cell immunity can occur during RHV persistence. Because CD8 T cells are required for vaccine clearance of RHV infection[13], it is highly likely that maintenance of responses to intact epitopes was a cause rather than consequence of viral elimination. How these responses were preserved is not yet clear, but their association with CD4 T cell help suggests that this interaction may be pivotal. During acute HCV infections, maintenance of an effective CD4 T helper response is a strong determinant of viral clearance[30–32], and loss of this subset is thought to underlie impaired control by HCV-specific CD8 T cells[33]. Viral silencing or inadequate priming of RHV-specific CD4 T cells by vaccine may thus ultimately drive persistence of escape virus. Indeed, in an earlier study evaluating a T cell-based vaccine in chimpanzees, control of heterologous HCV infection correlated with the quality of vaccine-elicited T helper immunity[22]. The recombinant human adenovirus vector utilized here is considered a poor inducer of CD4 T cell help[34] and, in a setting where CD8 T cells are already strained due to reduced recognition of virus, this factor may significantly undercut vaccine efficacy. Consequently, two potential solutions to improving vaccine efficacy in this model include increasing T cell breadth by inclusion of additional viral proteins (e.g. E1/E2) and bolstering CD4 T cell immunity via boosting with a heterologous viral vector such as MVA[21].

Vaccinated rats that previously controlled antigen-matched RHV infection were fully protected against secondary challenge with escape virus, except for a single animal that developed breakthrough viremia. The rate of protection exceeded that provided by vaccination alone, indicating a significant enhancement of RHV immunity not unlike that observed after spontaneous resolution of acute HCV infections in naïve subjects[35,36]. This effect could have been mediated by generation of *de novo* T cell responses specific to non-vaccine encoded antigens such as structural proteins[17] and/or qualitative enhancement of existing T cells that conferred lasting reactivity to intact epitopes. Indeed, inclusion of structural proteins has been implicated in improved RHV[15] and HCV vaccine efficacy[15]. An additional possibility is stimulation of an anti-E1/E2 neutralizing antibody response that helped suppress RHV viremia prior to T cell compromise. Investigation of the immune mechanisms that underlie this improved protection could provide a novel blueprint for inducing broadly effective HCV immunity.

Prior studies of HCV in humans suggest that unfit CD8 T cell escape mutations will revert under settings of reduced or absent selection pressure. This has been described after transmission into recipients mismatched at the restricting MHC allele[25,37,38] and following the immunoregulatory changes associated with pregnancy[18]. Stable reversion of RHV escape mutations in two different RT1-A^*l*^-restricted epitopes following passage into naïve rats provides a unique example of this phenomenon occurring in immune-competent, MHC-matched recipients. For reasons that have yet to be explained, RHV-specific CD8 T cells are severely dysfunctional during infection of naïve rats and consequently do not apply selective pressure against persistent virus[13,17]. Uniquely, emergence of RHV escape mutations has only been described when CD8 T cell pressure is induced via vaccine[13,15] or following acute antiviral treatment with DAA[17]. Interestingly, loss of the F199L substitution was not observed after virus passage, so reversion of RHV escape mutations to their ancestral state is not assured in every case. It will be interesting to determine in future studies whether and how new mutations acquired via serial passage of escape virus in vaccinated rats acquire lasting stability.

In conclusion, our results reveal antigen mismatch as an important determinant of RHV T cell vaccine success. Since HCV possesses a high degree of genetic variability, this factor may substantially weaken the efficacy of analogous vaccination approaches in humans. Further understanding of the mechanisms that facilitate heterologous RHV immunity, which appears possible, may have important implications for development of broadly effective HCV vaccines in humans, where similar studies are difficult to pursue.

## Materials and methods

### Ethics statement

All biohazard and animal experiments were carried out in accordance with approved protocols from the Nationwide Children’s Research Institute Institutional Biosafety Committee (protocol number IBS00000285) and the Institutional Animal Care and Use Committee (protocol number AR15-00116), respectively.

### Animals

Male and female Lewis rats were obtained from Charles Rivers Laboratories and bred under standard protocol. Rats were 6–9 weeks of age at time of study. All animal experiments were approved by the Institutional Animal Care and Use Committee by the Abigail Wexner Research Institute prior to initiation.

### Viruses and infections

The wild-type RHV inoculum was derived *in vivo* from a consensus genomic clone as described[14]. The escape variant described herein was isolated from a single vaccinated rat that developed persistent infection coincident with mutations in defined class I epitope sequences. For all infections, rats were challenged intravenously via tail vein with 10^6^ viral genomes.

### Virus quantification

RHV titers were determined exactly as described[13] with the lone modification that serum viral RNA was extracted using the Quick-RNA viral kit (ZYMO RESEARCH). In brief, viral cDNA was generated from serum extracted RNA using the GoScript reverse transcription kit (Promega) with random hexamer priming, followed by quantification on a StepOnePlus RT-PCR system (Applied Biosystems) using the PowerUP SYBR green master mix (Applied Biosystems). A standard curve was generated using a linearized plasmid encoding the RHV NS3 protein. The limit of detection of viral RNA was determined to be 1875 genomes/mL serum.

### Viral polyprotein sequencing

Overlapping PCR fragments spanning the entire RHV polyprotein coding sequence were amplified from viral cDNA using the Q5 High-Fidelity Polymerase (New England Biolabs) and directly sequenced (Eurofins Genomics) using nested sequencing primers. All non-synonymous changes in the consensus polyprotein sequence (accession no. KX905133) were analyzed for occurrence of mixed sequencing peaks and noted in the amino acid sequence where possible. The mixed I978V/T980S quasispecies variant was confirmed by standard clonal sequencing using the pGEM®-T easy vector (Promega). All primer sequences used are listed in Table 2.

**Table 2:** RHV primer sequences.

Name	Sequence (5‘➔3’)	Name	Sequence (5’➔3’)
C-NS2-1-F1	CGAGGCGTTTCCGCTGTAA	NS34-4-F1	GCTTGGAGGGGCCTTTC
C-NS2-1-F2	CGAGGCGTTTCCGCTGTAAACC	NS34-4-F2	CTTGGAGGGGCCTTTCAGG
C-NS2-1-R1	CACTTGCTCGCAGATGACACA	NS34-4-R1	GCAGCCCCAAAGACAGC
C-NS2-1-R2	CTTGCTCGCAGATGACACAGCC	NS34-4-R2	CCCAAAGACAGCAGCGCC
C-NS2-2-F1	CTGCACTGAGCTTTCCTGCAT	NS34-5-F1	AGCTCGCTGTCCCCTCT
C-NS2-2-F2	CTGCACTGAGCTTTCCTGCATGCA	NS34-5-F2	CTCGCTGTCCCCTCTCCC
C-NS2-2-R1	CAGTTGTYAGCCAGGGYGTAGT	NS34-5-R1	CCACCAGCATCTGCACCA
C-NS2-2-R2	GCCAGGGYGTAGTGCCACA	NS34-5-R2	CACCAGCATCTGCACCATGC
C-NS2-3-F1	GCATACCATGTGGGAGGCTT	NS5-1-F1	CCGGATACAAGGGACCTTG
C-NS2-3-F2	CATACCATGTGGGAGGCTTCGG	NS5-1-F2	CCGGATACAAGGGACCTTGGAA
C-NS2-3-R1	GCGAAGATCACAAGAGCTCC	NS5-1-R1	CCACATCGTACGTACCAGC
C-NS2-3-R2	GCGAAGATCACAAGAGCTCCAGC	NS5-1-R2	CCACATCGTACGTACCAGCCGT
C-NS2-4-F1	GAGGCTTACGAAGGCGG	NS5-2-F1	CCTCCCGTATGACACCGA
C-NS2-4-F2	GAGGCTTACGAAGGCGGTGA	NS5-2-F2	CCTCCCGTATGACACCGAGGA
C-NS2-4-R1	GAAGCGTGACAGGGGAGT	NS5-2-R1	CATCAAGCCGAATGTGTACGT
C-NS2-4-R2	AGCGTGACAGGGGAGTGCC	NS5-2-R2	CATCAAGCCGAATGTGTACGTGTG
NS34-1-F1	GGCAAGACGGTCAGAGC	NS5-3-F1	CAGCTTACACTCCAGCAGCTG
NS34-1-F2	GACGGTCAGAGCGGGCAT	NS5-3-F2	GCTTACACTCCAGCAGCTGGGA
NS34-1-R1	GCCTCAGGTATCTGGGC	NS5-3-R1	GTGCTGTCAAAGCACACGGT
NS34-1-R2	CCTCAGGTATCTGGGCTCC	NS5-3-R2	GTGCTGTCAAAGCACACGGTGTC
NS34-2-F1	CCTCCCGTATGACACCGA	NS5-4-F1	CTGTGTGCGGGGATGCATA
NS34-2-F2	GTGGTTAGCAGCCTGACAGG	NS5-4-F2	CTGTGTGCGGGGATGCATATGG
NS34-2-R1	CATCAAGCCGAATGTGTACGT	NS5-4-R1	GTGGGTTGTAGCCCTTTCC
NS34-2-R2	CATCCGTGGCAACGACGAC	NS5-4-R2	GTGGGTTGTAGCCCTTTCCCTC
NS34-3-F1	GCCAACGATCTGAGGGC	NS5-5-F1	CAGTGGCCATGAAGCGCAT
NS34-3-F2	CCAACGATCTGAGGGCAGC	NS5-5-F2	GTGGCCATGAAGCGCATGGG
NS34-3-R1	CGCCTGCAAGGGTCATAAC	NS5-5-R1	GGTGGTAAGAGTTGGAGGTTG
NS34-3-R2	CTGCAAGGGTCATAACCGTCTC	NS5-5-R2	GTGGTAAGAGTTGGAGGTTGAGGG

### Vaccinations

The recombinant human adenovirus serotype 5 vector expressing the RHV NS3-5B protein (Ad-NS) under control of a high-expression CMV promotor has been described[13]. Notably, the GDD catalytic site of the NS5B RNA polymerase was mutated to GNN to disrupt in vivo replication activity. The vector was generated and titrated by Vector Biolabs. Rats received two intramuscular doses of Ad-NS (5 x 10^8^ infectious units) separated three weeks apart. Three weeks after booster injection, rats were challenged with wild-type or escape virus.

### Peptides

All peptides were obtained from Genemed Synthesis as a lyophilized powder. 10 mg/mL stock solutions were prepared in a 10% DMSO-water solution and stored at -80° C until use. The final concentration of each peptide in all functional assays was 5 or 10 μg/mL unless otherwise specified.

### Leukocyte isolation, culture, and cryopreservation

Isolation and culture of liver-infiltrating leukocytes was performed exactly as described[13]. In brief, PBS-perfused livers were gently homogenized through a stainless-steel mesh in PBS supplemented with 2.5% FBS (Gibco). Cells were then isolated via 37% Percoll (GE Life Sciences) gradient density centrifugation at 500 x g for 20 min followed by lysis of residual RBCs in ACK buffer (Gibco). For cytokine stimulation assays described below, cells were cultured in RPMI-1640 containing GlutaMAX and HEPES (Gibco), 10% FBS (Gibco), 50 U/mL penicillin-streptomycin (Gibco), and 55 μM 2-mercaptoethanol (Gibco) at 37° C. For storage, cells were cryopreserved in FBS containing 10% DMSO via standard protocol.

### Antibodies and flow cytometry

The following rat reactive antibodies (clone, staining dilution) were obtained from BD Biosciences, eBioscience, and Miltenyi Biotec for use in flow cytometric analysis: CD3-VioGreen (REA223, 1:300), CD3-BV421 (1F4, 1:200), CD4-PerCP-eFluor710 (OX35, 1:250), CD8-BV786 (OX8, 1:100), IFNγ-AF647 (DB-1, 1:100), IFNγ-eFluor660 (DB-1, 1:50), TNFα-FITC (TN3-19.12, 1:50), and TNFα-PE (TN3-19.12, 1:100). Surface stains were performed for 20 min at 4° C in 50 μL PBS supplemented with 2.5% FBS (Gibco) and 0.1% sodium azide (Sigma Aldrich) in 96-well U-bottom plate. Dead cells were excluded from analysis using the LIVE/DEAD nearIR dye (Invitrogen) per manufacturer’s protocol. Events were collected on a BD Fortessa flow cytometer following compensation with UltraComp eBeads (Invitrogen). Data were analyzed using FlowJo v7.6.5 (Tree Star).

### Intracellular cytokine analysis

Quantification of T cell cytokine production by intracellular staining was performed as described[13]. In brief, 10^6^ cells were stimulated in a single well of a 96-well U-bottom plate with peptide for 5-hr in the presence of brefeldin-A, followed by intracellular cytokine staining using the Cytofix/Cytoperm kit (BD Biosciences). Stimulation with no peptide and PMA + Ionomycin (BioLegend) were used as negative and positive controls, respectively. Response magnitude was calculated by subtracting the frequency of cytokine positive cells in negative control from that of experimental samples. A positive response was defined as ≥3 times background staining prior normalization.

### Tetramer staining

Biotinylinated rat RT1-A^*l*^ monomers specific for the RHV E1_191_ and NS5B_2511_ epitopes were obtained from the NIH Tetramer Core Facility and tetramerized with streptavidin-APC (Prozyme) via standard protocol. For direct detection of virus-specific CD8 T cell populations, liver-infiltrating leukocytes were stained for 30 min at 4° C (1:100) with tetramer alone followed by surface labeling with anti-rat CD3 and CD8 as above. Following staining with LIVE/DEAD near IR dye (Invitrogen), cells were fixed in 1% PFA prior to analysis.

### Statistics

Differences in vaccine outcome were assessed for significance using Fisher’s exact test. Differences in T cell immunity were assessed for significance using Student’s t-test with unequal variance.

## Supporting information

S1 Fig Evolution of RHV polyprotein in vaccinated rat R558. Overlapping PCR fragments spanning the complete RHV coding segment were amplified by a high-fidelity polymerase and directly sequenced. Consensus amino acid substitutions are shown. Shaded sequences mark location of known RT1-A^*l*^-restricted class I epitopes. Multiple sequencing peaks were resolved where possible into mixed amino acid residues.
(DOCX)
