# Supplementary material for: Adenovirus-vectored T cell vaccine for hepacivirus shows reduced effectiveness against a CD8 T cell escape variant in rats
Source: PLoS Pathog. 2021 Mar 18;17(3):e1009391. doi: 10.1371/journal.ppat.1009391 (PMC8009437; doi:10.1371/journal.ppat.1009391)
Supplement: S1 Fig — Overlapping PCR fragments spanning the complete RHV coding segment were amplified by a high-fidelity polymerase and directly sequenced. Consensus amino acid substitutions are shown. Shaded sequences mark location of known RT1-Al-restricted class I epitopes. Multiple sequencing peaks were resolved where possible into mixed amino acid residues. (DOCX) [file ppat.1009391.s001.docx]

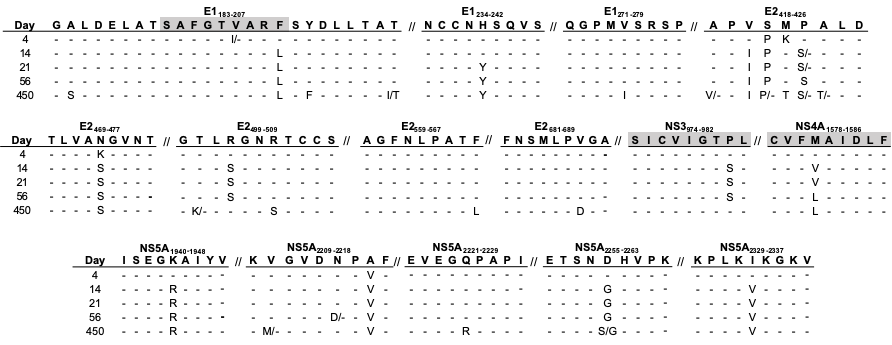


**Figure S1. Evolution of RHV polyprotein in vaccinated rat R558.** Overlapping PCR fragments spanning the complete RHV coding segment were amplified by a high-fidelity polymerase and directly sequenced. Consensus amino acid substitutions are shown. Shaded sequences mark location of known RT1-A*^l^*-restricted class I epitopes. Multiple sequencing peaks were resolved where possible into mixed amino acid residues.
